# Stimulating the Lip Motor Cortex with Transcranial Magnetic Stimulation

**DOI:** 10.3791/51665

**Published:** 2014-06-14

**Authors:** Riikka Möttönen, Jack Rogers, Kate E. Watkins

**Affiliations:** ^1^Department of Experimental Psychology, University of Oxford

**Keywords:** Behavior, Issue 88, electromyography, motor cortex, motor evoked potential, motor excitability, speech, repetitive TMS, rTMS, virtual lesion, transcranial magnetic stimulation

## Abstract

Transcranial magnetic stimulation (TMS) has proven to be a useful tool in investigating the role of the articulatory motor cortex in speech perception. Researchers have used single-pulse and repetitive TMS to stimulate the lip representation in the motor cortex. The excitability of the lip motor representation can be investigated by applying single TMS pulses over this cortical area and recording TMS-induced motor evoked potentials (MEPs) via electrodes attached to the lip muscles (electromyography; EMG). Larger MEPs reflect increased cortical excitability. Studies have shown that excitability increases during listening to speech as well as during viewing speech-related movements. TMS can be used also to disrupt the lip motor representation. A 15-min train of low-frequency sub-threshold repetitive stimulation has been shown to suppress motor excitability for a further 15-20 min. This TMS-induced disruption of the motor lip representation impairs subsequent performance in demanding speech perception tasks and modulates auditory-cortex responses to speech sounds. These findings are consistent with the suggestion that the motor cortex contributes to speech perception. This article describes how to localize the lip representation in the motor cortex and how to define the appropriate stimulation intensity for carrying out both single-pulse and repetitive TMS experiments.

**Figure:**
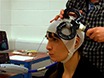


## Introduction

Speech perception is a demanding skill that requires encoding of complex and highly variable incoming auditory signals. Whilst it is uncontroversial that the auditory cortex plays a critical role in speech processing, whether the motor regions that control the movements of the articulators (*e.g.* lips) during speech production also contribute to speech perception remains a topic of active investigation and scientific debate^1-5^. The idea that motor representations are involved in speech perception is not novel. According to Liberman's motor theory of speech perception^6,7^ the listener perceives speech by simulating the "intended articulatory gestures" of the speaker. TMS has proven to be a powerful tool in investigating the putative role of the articulatory motor cortex during speech perception (for a review, see ^8^). This article focuses on stimulation of the lip motor representation using single-pulse and repetitive TMS techniques.

Single-pulse TMS has provided a highly effective means of investigating the link between the motor cortex and speech processing^8-10^. Single TMS pulses applied to the primary motor cortex (M1) elicit motor evoked potentials (*i.e.* MEPs) in the contralateral target muscle that can be recorded using electromyography (EMG). MEPs recorded from the hand muscles (first dorsal interosseus; FDI) peak approximately 24 msec after the pulse, whereas MEPs recorded from the lip muscles (orbicularis oris; OO) peak approximately 15 msec after the pulse (see **Figure 1**). This reflects differences in the distance from the motor cortex to the lips and hand muscles; the cortico-bulbar pathway to the lips is shorter than the cortico-spinal pathway to the hands. The intensity of the pulse required to elicit an MEP differs greatly across participants, most likely reflecting neuroanatomical differences and differences in the skull thickness^11^. The amplitude of the MEP is dependent on the functional state of the motor system, with pulses of a constant intensity inducing larger MEPs when the target muscle is contracted compared to when the muscle is relaxed. MEP measurements provide an accurate means of localizing the cortical representations of different muscles in each participant as well as normalizing the TMS intensity between subjects. This method also provides a direct measure (*i.e.* MEP amplitude) of motor excitability relative to an independent variable of interest. For instance, single-pulse TMS studies have shown that stimulation of the lip-representation induces larger MEPs (*i.e.* increased excitability) when listening to speech and viewing speech-related lip movements^10,12,13^. A combined PET and single-pulse TMS study showed that the excitability of the speech motor system during auditory speech perception is modulated in part by activity in the posterior left inferior frontal gyrus^14^.

Whilst single-pulse TMS can demonstrate changes in the excitability of the motor system during speech perception it does not indicate whether the motor cortex contributes to speech processing. Repetitive TMS (rTMS) can be used to induce a temporary disruption (*i.e.* "a virtual lesion") in the motor cortex^15^. This "virtual lesion" approach enables investigation of speech perception during controlled, short-term disruption to a focal area of the motor system. The "virtual lesions" caused by TMS differ from real lesions in patients which often cover widespread cortical regions leading to functional reorganization of the brain over time. Patient studies typically compare behavior of the patients to a control group and rarely provide knowledge of performance prior to the stroke/lesion. By using rTMS it is possible to investigate a participant's ability to perform speech perception tasks with and without motor disruptions and, therefore, examine whether these disruptions contribute to performance.

Sub-threshold low-frequency rTMS can be used to disrupt the motor lip representation temporarily and it has been used to investigate the role of the articulatory motor cortex in speech perception^16-18^. In these experiments, monophasic pulse sequences were used because monophasic low-frequency rTMS has been shown to be more effective in decreasing cortical excitability compared to biphasic rTMS^19^. The size of MEPs recorded from the lips decreases after a 15-min train of sub-threshold monophasic TMS pulses applied at rates below 1 Hz *i.e.* motor excitability is suppressed^18^. This rTMS-induced disruption also impairs listeners' ability to categorically perceive acoustic continua ranging between two speech sounds that differed in place of articulation (*e.g.* 'ba' vs. 'da' and 'pa' vs. 'ta'). The impaired performance after disruption to the lip motor cortex suggests that the motor system contributes to speech perception. Disruption of the hand motor representation has no effect on categorical perception of speech sounds. These findings are consistent with earlier findings showing that low-frequency rTMS applied to premotor cortex impairs performance in a phonetic discrimination task when using syllables presented in noise compared to a color discrimination control task matched on difficulty, task structure and response type^20^. These studies demonstrate that rTMS is an extremely effective means of exploring the auditory-motor circuits that may support both speech production and perception. Low-frequency rTMS can also be used in conjunction with neuroimaging techniques to further investigate this issue (see Discussion).

## Protocol

### 1. Preparation

1. Ask the participant to fill out a safety screening form (see *e.g.* ^21^). Note: Participants who have contraindications for TMS should not be stimulated; the most common contraindications are: lack of sleep, medication (*e.g.* antidepressants), and a family history of epilepsy.
2. Explain the TMS procedure and the experimental details to the participant and obtain informed consent.
3. Using alcohol, clean the skin above the belly of the FDI muscle in the right hand and in the reference site (*e.g.* tendon of the FDI muscle) and attach electrodes on these sites.
4. Clean the skin on the right side of the OO muscle with alcohol and attach one electrode to the right corner of the upper lip and one electrode to the right corner of the lower lip.
5. Clean the skin around the site for the ground electrode (*e.g.* on the forehead) and attach the electrode.
6. Connect the wires of electrodes into an electrode box attached to an EMG recording system.
7. Check the EMG signals recorded from the hand and lip muscles when the participant is contracting and relaxing these muscles (by visually inspecting them using *e.g.* Spike2 software). If the signals look noisy when the participant relaxes the lip and hand muscles, twist the electrode cables, re-clean the skin at the electrode site and/or ask the participant to uncross their legs, remove their shoes and have their feet on the floor (better grounding).
8. Protect the participant's hearing by using earplugs.
9. Put the cap on the participant's head in order to be able to mark the position of the TMS coil.

### 2. Localization of Motor Hand Representation

1. Mark the vertex on the cap (by attaching a little sticker or by using a pen) and measure the distance from the vertex to the left preauricular point. Move 33% of this distance from the vertex towards the left preauricular point and mark this spot.
2. Place the center of the figure-of-eight TMS coil on this spot. Orient the handle of the coil 45 degrees from the midline.
3. Deliver the first TMS pulse *e.g.* by pressing a foot pedal. Choose a low intensity (*e.g.* 40% of the max stimulator intensity) if the participant's motor threshold is not known. Move the coil slightly and/or increase the intensity, if no MEP or muscle twitch is visible in the hand.
4. When an MEP is elicited, keep moving the coil in 5-mm steps around this area in order to find an appropriate "hot spot", *i.e.* the site and coil orientation that elicits the maximal MEPs at a certain intensity. Keep at least a 5-sec break between the pulses. Observe the participants hand to check which muscles twitch. When the spot in which the MEPs are the largest is found, mark this "hot spot" and the orientation of the coil on the cap.

### 3. Localization of Motor Lip Representation

1. Mark a spot 2-3 cm from the FDI spot along a straight line towards the corner of the left eye (the location of the motor representation is more anterior and inferior than that of the hand representation).
2. Place the coil on this spot. Ask the participant to purse the lip muscles; this lowers the motor threshold and, therefore, makes it easier to find the motor lip representation.
3. Tell the participant that the TMS pulses may feel more intense in this location than in the previous location and that s/he may feel twitches in his/her face (due to peripheral stimulation) and involuntary eye-blinks. Ask the participant to inform the experimenter at any point if the stimulation becomes unpleasant or painful or if they wish to stop the stimulation.
4. Deliver the first pulses. If no MEPs are elicited, move the coil slightly and/or increase the intensity. Keep at least a 5-sec break between the pulses. When an MEP is elicited, keep moving the coil around this area in 5-mm steps in order to find a "hot spot" for the OO muscle. Note: The shape of the lip MEPs is often multiphasic and their shape can vary from participant to participant. Also, the motor threshold is often higher for the lip muscle than for the hand muscles.

### 4. Defining Intensity for Single Pulse Experiments

1. Train the participant to maintain a constant level of contraction, if MEPs will be recorded from contracted lip muscles. Note: Providing visual feedback about the power of the EMG signal (*e.g.* by using Spike2 software) helps during the training; training can be stopped when the participant is able keep the level of contraction stable for at least 1 min.
2. Place the coil on the hot spot for the OO muscle. Deliver 10 pulses with fixed intensity. Keep at least a 5-sec break between the pulses. Estimate the sizes of MEPs by visually inspecting them. Increase the intensity, if MEPs are very small or there was no MEP on every trial. Deliver 10 pulses again. Keep increasing the intensity until a robust MEP is elicited on every trial (*e.g.* with amplitude of approximately 0.3 mV when the lip muscle is relaxed or with amplitude of approximately 1 mV when the lip muscle is contracted). Use this intensity during the single-pulse experiment. Note: It is a good practice to report stimulator intensities in publications.

### 5. Defining Active Motor Threshold for rTMS Experiments

1. Ask the participant to contract the lip muscles as hard as they can. Determine the amplitude of this maximal contraction (by visually inspecting the EMG signal).
2. Ask the participant to reduce the contraction of the lips. Guide him/her to reach the contraction level that is roughly 20% of the maximum. Ask the participant to keep this level for 1 min. Give him/her a short break and repeat the practice as many times as needed. Note: Providing visual feedback about the power of the EMG signal (*e.g.* by using Spike2 software) helps during the training; training can be stopped when the participant is able keep the level of contraction stable for at least 1 min.
3. Deliver 10 pulses over the hotspot for OO while the participant is contracting the lip at 20% of the maximum. Count how many MEPs were elicited. If there was an MEP (with peak-to-peak amplitude of at least 0.2 mV) in fewer than 5 out of 10 trials, increase the intensity. If there was an MEP on more than 5 out of 10 trials, lower the intensity. Repeat until a minimum intensity level that elicits MEPs on at least 50% of the trials (active motor threshold) has been found.
4. Ask the participant to relax the lip muscles and deliver 10 pulses at the intensity of active motor threshold. Check that no MEPs were elicited (*i.e.* stimulation intensity is sub-threshold). If no MEPs were elicited, use this intensity (*i.e.* 100% of active motor threshold) during the rTMS train (while the lip muscle is relaxed). If MEPs were elicited, go back to 5.3.

### 6. Low-frequency rTMS

1. Deliver TMS pulses at a frequency of up to 1 Hz for 15-min during the rTMS train. When using the MagStim BiStim system that consists of two stimulators, trigger these stimulators alternatingly (*i.e.* each stimulator delivers a pulse every 3 sec.) in order to create a monophasic pulse sequence of 0.66 Hz. Note: Spike2 software together with the EMG acquisition system (by Cambridge Electronics) can be used to create a sequence of trigger pulses. Note: In practice, the maximum intensity for 0.66 Hz pulse train using the MagStim BiStim system and standard coils is 65% of the maximum stimulator output. This limit relates to the maximum pulse frequency of each stimulator (0.33 Hz), over-heating of the coils and participant comfort.
2. Monitor recordings from the lip and the hand muscles during the rTMS train to ensure no MEPs are elicited that would indicate an increase in excitability or spreading effects to the neighboring representation. Also monitor the participant for signs of discomfort or changes in the level of alertness.
3. Change the coil after 7.5 min in order to avoid overheating. Note: This step can be omitted when using special coils that are cooled during the stimulation; heating of the coils can affect the strength of the magnetic field.

## Representative Results

### Results from single pulse experiments

In single pulse experiments, the dependent measure is the MEP amplitude. The size of the MEPs is typically measured either as peak-to-peak amplitude^13,18^ or area under the curve^10^. The lip MEPs can be recorded from either the relaxed muscle or a slightly contracted muscle. In the latter case, the TMS pulses can be delivered with a lower intensity, because the contraction lowers the motor threshold. It is very important that the level of the contraction stays constant throughout the experiment, because the strength of the contraction affects the MEP amplitudes. The stronger the contraction is, the larger the MEPs are. Therefore, it is important to train the participant to maintain a constant level of contraction before defining the TMS intensity, if MEPs are recorded from the contracted muscle. Visual feedback helps during the training (see Protocol 4.1.). Sometimes the threshold is so high that the intensity of the TMS pulses is uncomfortable for the participant and the experiment cannot be carried out. Also, it is not always possible to find the lip representation or record robust MEPs, especially when the lip muscles are relaxed. It is a good practice to report the number of participants in whom the experiment could not be carried out in publications. **Figure 1** shows MEPs recorded from a relaxed and contracted lip muscle for a single participant. The intensity of the TMS pulses was kept constant across three levels of contraction. The motor excitability increases when the muscle is contracted, and consequently the MEPs get larger.


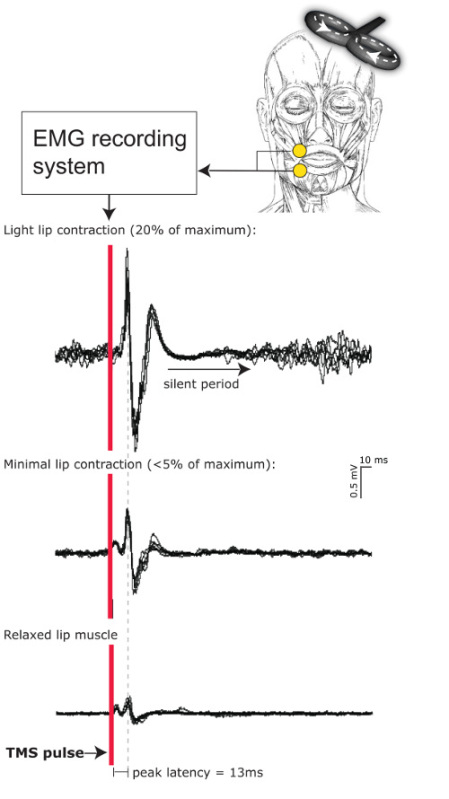
**Figure 1. The effect of muscle contraction on lip MEPs.** The MEPs were measured from one participant while she (1) relaxed her lips, (2) contracted the lips as slightly as she could (< 5% of the maximum), and (3) when she contracted the lips about 20% of the maximum. The intensity of the mono-phasic TMS pulses was the same in all three conditions (58% of the maximum intensity). 6 MEPs were recorded in each condition (overlaid in the figure). The figure illustrates that the MEPs get larger when the level of contraction increases. A cortical silent period is clearly visible in the condition with strongest contraction. Please click here to view a larger version of this figure.

Motor excitability of the lip representation increases during listening to speech and viewing speech-related lip movements. **Figure 2** shows lip MEP recorded during listening to speech and nonverbal noise, and during watching eye movements and speech related lip movements^10^. In this study MEPs were recorded from slightly contracted lip muscles. The level of the contraction was added as a covariate in the MEP analysis and used to adjust the MEP size. The lip MEPs elicited by left M1 stimulation were significantly enhanced during listening to speech and watching speech-related lip movements relative to the baseline condition, whereas the lip MEPs elicited by right M1 stimulation were not modulated during any of the conditions.


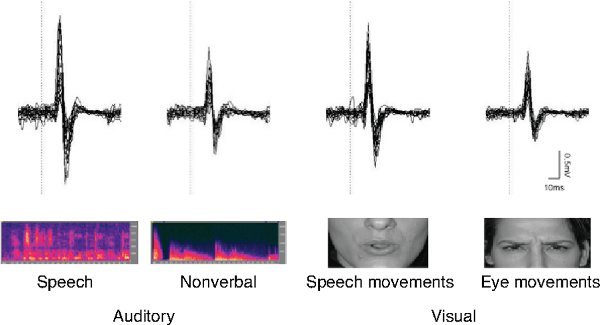
**Figure 2. MEPs during perception of auditory and visual speech in one participant.** The MEPs were recorded from the slightly contracted lips muscle while the left motor cortex was stimulated. The MEPs were enhanced during listening to speech and viewing speech related lip movements. Figure modified from ^10^.

A recent study investigated the specificity of changes in excitability in the lip motor cortex during observation of visual mouth movements^13^. Z-scores for lip MEPs recorded during visual perception of known speech (English), unknown speech (Hebrew), non-speech mouth movements (gurning) and a still face are presented in **Figure 3**. These z-scores were calculated relative to the mean of all conditions. TMS pulses were delivered over the left M1 and MEPs recorded from the relaxed lip muscle. MEPs were larger during observation of known speech than unknown speech or non-speech mouth movements. The MEPs recorded during observation of a still face were as large as during observation of English speech. These findings suggest that the lip motor cortex participates in processing of visual signals during speech communication. Please click here to view a larger version of this figure.


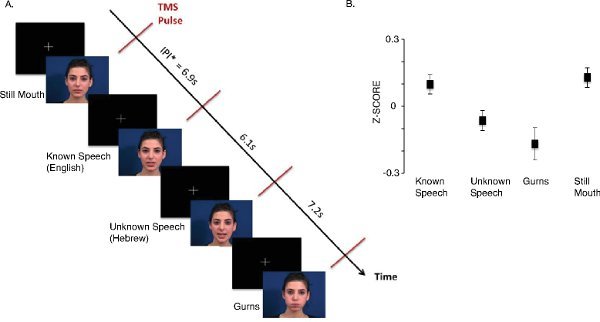
**Figure 3. Motor excitability during perception of visual speech.A.** Participants were presented videos of known speech (*i.e.* English), unknown speech (*i.e.* Hebrew), non-speech mouth movements (*i.e.* gurns) and a still mouth. One TMS pulse was delivered during each video. Inter-pulse-interval (IPI) varied between 5 and 8 seconds. **B.** The figure shows****standardized amplitudes of MEPs (± SEM) measured from the lip during observation of videos. The z-scores were calculated relative to the mean of all conditions. The MEPs were significantly larger during observation of known speech than unknown speech (p = 0.001) or gurns (p < 0.05). Differences in MEP amplitudes between conditions reflect differences in the excitability of the lip representation in the motor cortex. Figure modified from^13^. Please click here to view a larger version of this figure.

### Results from rTMS experiments

It had been shown that low-frequency rTMS over the hand motor representation can reduce the motor excitability and induce a temporary disruption in this area (*i.e.* "a virtual lesion")^15^. rTMS over the lip motor representation also reduces excitability of this area^18^. Changes in MEPs amplitudes after 15-min of low-frequency stimulation over the lip representation in the left M1 cortex are shown in **Figure 4**. The MEPs recorded from the lips were suppressed 7 min after the end of the repetitive TMS train, but had started to recover 15 min after. This suppressed excitability shows that low-frequency rTMS disrupted functioning of the lip representation in the motor cortex for about 15 min.

The TMS-induced disruptions in the articulatory motor cortex impair participants' performance in the demanding speech perception tasks. **Figure 5** shows how TMS-induced disruption of the lip representation modulated performance in a same-different discrimination task^18^. The participants were presented with pairs of synthetic syllables both before low-frequency rTMS and after it. Their task was to indicate whether syllables were the same or different. The TMS-induced disruption impaired the participant's ability to discriminate synthetic speech sounds that are lip-articulated from speech sounds that are not articulated by the lips ('ba' vs. 'da' and 'pa' vs. 'ta'). However, this disruption did not influence their ability to discriminate two speech sounds that are not articulated by the lips ('ka' vs. 'ga' and 'da' vs. 'ga'). This suggests that the lip representation contributes to speech perception in an articulator specific manner.


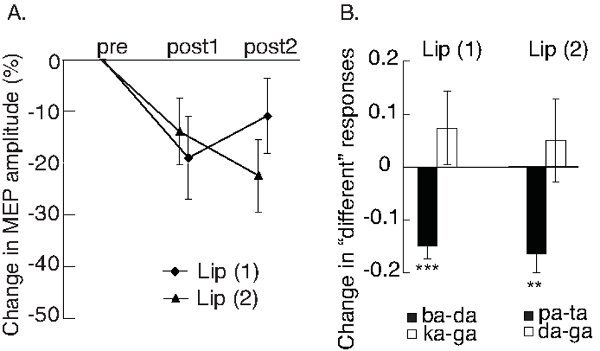
**Figure 4. Effects of rTMS on motor excitability and speech discrimination. A.** The graph presents mean changes (± SEM) in peak-to-peak amplitudes of post-rTMS MEPs in relation to pre-rTMS MEPs. The MEPs were recorded from the lip muscles and the rTMS was applied over lip motor cortex in left hemisphere in both experiments 1 and 2. The post-rTMS MEPs were recorded ~7 min (post1) and ~15 min (post2) after the end of the 15-min low-frequency rTMS train. MEPs were significantly suppressed after rTMS in both experiments 1 and 2. **B.** Participants were presented with synthetic speech sounds from eight-step acoustic continua between two speech sounds. The "across-category" pairs were selected based on the place of category boundaries that were determined for each participant individually. The participants performed same-different discrimination task pre and post low-frequency rTMS over the lip motor representation. Changes in proportions of "different" responses (± SEM) are plotted. After TMS, participants were poorer in discriminating across-category pairs that included lip-articulated speech sounds ('ba' vs. 'da' and 'pa' vs. 'ta') than before rTMS. Discriminability of other pairs stayed stable. Figures are modified from^18^. **p < .01, ***p < .001. Please click here to view a larger version of this figure.

## Discussion

During the past two decades, TMS has become a widely used method in cognitive neuroscience, because it can provide information that is complementary to neuroimaging. It has also provided speech scientists with new tools to investigate whether the motor speech production system could be involved in speech perception. Specifically, TMS provides an important means of testing experimentally whether the neural circuits that control speech articulation are facilitated whilst listening to speech and, whether these circuits contribute to speech perception.

This article described how the motor lip representation can be stimulated using TMS, and how both single-pulse and repetitive TMS techniques have been used to examine the role of the motor cortex in speech perception. The studies reported here provide evidence that the motor cortex contributes to speech processing in the human brain. Other TMS paradigms have also been used to investigate speech processing in the motor system. Dual TMS pulses delivered over the motor lip and tongue representations before auditory syllables have been shown to facilitate recognition of lip- and tongue-articulated syllables, respectively^22^. A paired-coil paradigm can be used to investigate effective connectivity between the lip representation and other cortical regions during speech perception^23^. It was shown that the effective connectivity between the motor lip representation and temporo-parietal junction and the inferior frontal cortex is enhanced during listening to speech, but not during listening to white noise. Continuous theta-burst stimulation (cTBS) over the temporo-parietal junction abolished its effective connectivity with the motor lip representation, providing further evidence that these cortical regions are functionally coupled during listening to speech^23^. The advantage of cTBS over low-frequency rTMS is that a relatively short train of cTBS (*e.g.* 40 sec) can produce a long-lasting disruption in the motor cortex (up to 60 min)^24^. However, the effects of cTBS on motor excitability are highly variable across participants^25^.

Combining TMS with other neuroimaging techniques that measure whole brain activity can provide insight into how TMS affects neural processes in both neighboring and more distal cortical regions. Brain regions undoubtedly interact with one another during perceptual and cognitive processes, and so it is unsurprising that an induced "virtual lesion" in one brain area would modulate functioning of other brain areas engaged in the same process. In order to advance understanding of the neural basis of speech perception, it is essential to investigate how the articulatory motor cortex interacts with the auditory regions in the superior temporal cortex during listening to speech and how these interactions contribute to speech perception. The combination of TMS with brain imaging techniques provides a means to address these questions. For example, it is possible to examine influences of TMS-induced disruptions in the articulatory system on processing of speech signals in the superior temporal cortex using electroencephalography (EEG), magnetoencephalography (MEG), functional MRI and positron emission tomography (PET). Experiments combining low-frequency rTMS and EEG demonstrate that TMS-induced disruption in the articulatory motor cortex modulates automatic discrimination of speech, but not non-speech, sounds in the auditory system, showing that these systems interact during speech processing^16^. Combination of rTMS with MEG is also a powerful method to investigate the timing of auditory-motor interactions^17^.

Nevertheless, the link between speech production and perception is still poorly understood. TMS combined with speech tasks and additional neuroimaging techniques can help scientists to improve understanding of the neural bases of speech perception and production and whether they overlap.

## Disclosures

The authors have nothing to disclose.
